# Spatiotemporal profile of Map2 and microglial changes in the hippocampal CA1 region following pilocarpine-induced status epilepticus

**DOI:** 10.1038/srep24988

**Published:** 2016-05-04

**Authors:** Nicole D. Schartz, Seth A. Herr, Lauren Madsen, Sarah J. Butts, Ceidy Torres, Loyda B. Mendez, Amy L. Brewster

**Affiliations:** 1 Department of Psychological Sciences, West Lafayette, IN 47907, USA; 2 School of Science and Technology, Universidad del Este, Carolina, PR 00984, Puerto Rico; 3 Weldon School of Biomedical Engineering, West Lafayette, IN 47907, USA

## Abstract

Status epilepticus (SE) triggers pathological changes to hippocampal dendrites that may promote epileptogenesis. The microtubule associated protein 2 (Map2) helps stabilize microtubules of the dendritic cytoskeleton. Recently, we reported a substantial decline in Map2 that coincided with robust microglia accumulation in the CA1 hippocampal region after an episode of SE. A spatial correlation between Map2 loss and reactive microglia was also reported in human cortex from refractory epilepsy. New evidence supports that microglia modulate dendritic structures. Thus, to identify a potential association between SE-induced Map2 and microglial changes, a spatiotemporal profile of these events is necessary. We used immunohistochemistry to determine the distribution of Map2 and the microglia marker IBA1 in the hippocampus after pilocarpine-induced SE from 4 hrs to 35 days. We found a decline in Map2 immunoreactivity in the CA1 area that reached minimal levels at 14 days post-SE and partially increased thereafter. In contrast, maximal microglia accumulation occurred in the CA1 area at 14 days post-SE. Our data indicate that SE-induced Map2 and microglial changes parallel each other’s spatiotemporal profiles. These findings may lay the foundation for future mechanistic studies to help identify potential roles for microglia in the dendritic pathology associated with SE and epilepsy.

Epilepsy is a neurological disorder characterized by spontaneous recurrent seizures. Evidence from experimental models support that episodes of prolonged and uninterrupted seizure activity (status epilepticus; SE) increase the risk for the generation of future unprovoked recurrent seizures (epilepsy)^1^. Mechanisms underlying neuronal hyperexcitability after SE include injury to the hippocampal network where dendrites are vulnerable to structural and morphological alterations^2^,^3^,^4^. SE-induced decreases in spine densities and dendritic branching are often observed in parallel to epileptogenesis and also are associated with the development of hippocampal-dependent cognitive deficits^2^,^3^,^5^,^6^.

Dendritic structural plasticity is in part controlled by cytoskeletal components such as those from the family of microtubule associated protein 2 (Map2) along with their phosphorylation status^7^,^8^,^9^. Map2 associates with microtubules to provide support to the neuronal cytoskeleton and is important for dendritic structural stability and neurite growth^7^,^8^,^9^. In addition, Map2 is essential for cellular functions such as the integration of synaptic inputs, local signal transduction, protein trafficking, and synaptic plasticity^7^,^9^,^10^,^11^. In both human and experimental models of epilepsy, decreases in cortical and hippocampal Map2 immunoreactivity (IR), along with Map2 dephosphorylation, have been reported^5^,^12^,^13^,^14^. While it is likely that a degree of Map2 loss is attributable to the neuronal death that is often seen after SE and in epilepsy^15^, altered Map2 levels in remaining neurons may impact the dynamics of dendritic structures. In order to understand the potential contribution that SE-induced Map2 changes may have on hippocampal dendritic stability, it is important to define their spatiotemporal evolution. To our knowledge, spatiotemporal analyses of SE-induced Map2 changes have only been reported in the developing brain^16^. Therefore, the first objective of this study is to characterize the temporal profile of the distribution of Map2 in the hippocampus of mature animals after an episode of SE.

Previously, we reported a substantial decline of Map2 IR in the hippocampal CA1 region after an episode SE that correlated with a robust accumulation of hypertrophied microglial cells^5^, the resident immune cells of the brain. Interestingly, following treatment with the drug rapamycin, an inhibitor of the mechanistic target of rapamycin (mTOR), the SE-induced Map2 and microglial changes were largely attenuated^5^. Because rapamycin modulates microglial properties in epilepsy^5^,^17^,^18^ as well as other injury models^19^,^20^,^21^, these data suggest that microglia may contribute to the observed Map2 dysregulation. Extensive evidence support microglia activation in the hippocampus in association with seizures in human and experimental models^5^,^22^,^23^,^24^,^25^,^26^,^27^,^28^,^29^. Interestingly, recent studies exposed new roles for microglia in the modulation of dendritic and axonal structures including synapse pruning^30^,^31^,^32^,^33^,^34^,^35^. Given the potential impact that SE-induced microglial changes may have on hippocampal dendritic stability, a second objective of this study is to characterize the spatiotemporal progression of SE-induced microglial changes in parallel to Map2. Therefore, here we used immunohistochemistry to assess the distribution of Map2 and the microglia marker IBA1 in the hippocampal formation of mature rats at various time points (4 hrs to 35 days) after an episode of pilocarpine-induced SE.

## Results

### SE triggers a transient decrease in Map2 immunoreactivity in the CA1 hippocampal area

**Figure 1:**
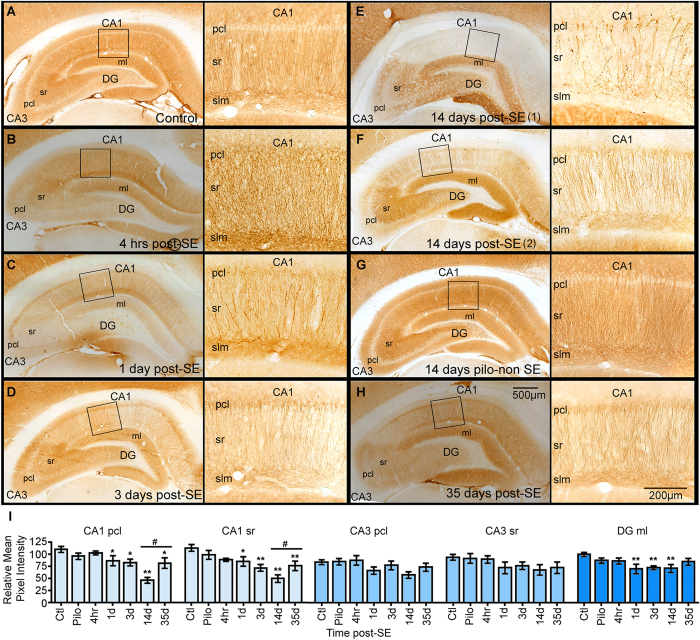
Temporal profile of Map2 immunostaining in the hippocampus after status epilepticus (SE). (**A**) shows a representative image of the Map2 staining (brown) from a control hippocampus with a high magnification image of the CA1 area (boxed). (**B–H**) show representative hippocampal images with high magnification of boxed CA1 areas at different time points after an episode of SE ((**B**)4 hrs; (**C**) 1 day (d); (**D**) 3d; (**E,F**) 14d; (**H**) 35d). A representative image of a hippocampus from a rat that was given pilocarpine but failed to develop SE (pilo-non SE; Pilo in graphs) is shown in (**G**). (**I**) shows the densitometry analysis as relative mean pixel intensity for the different hippocampal sub-regions CA1 pyramidal cell layer (pcl) and stratum radiatum (sr), CA3 pcl and sr, and the molecular layer (ml) of the dentate gyrus (DG). Note that significant differences in the intensity of Map2 immunoreactivity are evident within the CA1 region between the control group and 1–35d post-SE groups. Data are shown as mean ± standard error of the mean. *p < 0.05, **p < 0.01 compared to the control group. ^#^*p* < 0.05, comparison between 14d and 35d groups (n = 3–9/group). ANOVA with Fishers LSD post hoc test.

We determined the temporal progression in the distribution of Map2 immunostaining in the mature hippocampal formation at 4 hrs, 1-, 3-, 14-, and 35-days after an episode of pilocarpine-induced SE (Fig. 1). Densitometry analysis followed by Analysis of Variance (ANOVA) revealed significant group effects for the intensity of Map2 IR signal in the CA1 pyramidal cell layer (pcl) and stratum radiatum (sr) [CA1 pcl, *F* (6, 39) = 6.40, *p* < 0.01; CA1 sr, *F* (6, 39) = 6.12, *p* < 0.01], as well as the molecular layer (ml) of the Dentate Gyrus (DG) [DG ml, *F* (6, 39) = 3.35, *p* < 0.01] (Fig. 1I). In contrast, the intensity of Map2 IR localized in the CA3 region was not significantly altered by SE (4 hrs to 35 days) when compared to the control group (CA3 pcl [*F* (6, 39) = 1.79, *p* = 0.13], CA3 sr [*F* (6, 39) = 1.26, *p* = 0.30]). In control hippocampi, a homogenous distribution of Map2 IR was evident throughout the dendritic fields of CA1-3 sr (Fig. 1A). High magnification images of the CA1 area showed a continuous Map2 staining pattern within the labeled dendritic structures of control hippocampi (Fig. 1A). At 4 hrs post-SE, the intensity of Map2 IR over the CA1 pcl and sr regions was comparable to that of the control group (pcl, *p* = 0.61; sr, *p* = 0.10) (Fig. 1B). Between 1 and 14 days post-SE, the Map2 signal was gradually and significantly less intense than the control group in the CA1 pcl (ctl vs 1d, *p* = 0.04; ctl vs 3d, *p* = 0.01; ctl vs 14d, *p* < 0.01) and sr (ctl vs 1d, *p* = 0.02; ctl vs 3d, *p* < 0.01; ctl vs 14d, *p* < 0.01) regions (Fig. 1C–F). Note that a number of dendrites displayed prominent punctated Map2 staining by day 14. The significant decrease in Map2 IR in the CA1 area seen at two weeks post-SE was specific to the SE event. Rats that received the same dose of pilocarpine but did not develop class 5 seizures showed a hippocampal distribution of Map2 similar to controls (14 days pilo-non SE; Fig. 1G) (CA1 pcl, *p* = 0.61; CA1 sr, *p* = 0.10). Furthermore, the SE-induced decrease in Map2 IR was evident throughout the dorsoventral axis of the hippocampus (Supplementary Fig. 1). At 35 days following SE onset, the Map2 IR levels in CA1 pcl were significantly decreased compared to controls (*p* = 0.01) (Fig. 1H) and resembled that observed at 1–3 days post-SE (1d vs 35d, *p* = 0.67; 3d vs 35d, *p* = 0.92). However, a significant increase in the intensity of Map2 IR was observed between 14 and 35 days after SE (*p* < 0.01) suggesting a partial recovery of Map2 IR in the CA1 region.

Because phosphorylation of Map2 is an important modification that regulates microtubule assembly and dendritic stability^8^, we investigated the distribution of phosphorylated Map2 at the time points when total Map2 levels were significantly decreased (3-, 14- and 35-days post-SE; Fig. 1) (Supplementary Fig. 2). ANOVA revealed a group effect for the intensity of phospho-Map2 IR [*F* (4, 34) = 2.79, *p* = 0.04]. We found a significant reduction in the intensity of phospho-Map2 IR over the CA1 region at all time points after SE relative to the control group (ctl vs 3d, *p* = 0.02; ctl vs 14d, *p* = 0.01; ctl vs 35d, *p* < 0.01). This finding is consistent with a previous study showing that in human and experimental epilepsy dephosphorization of Map2 occurred in association with epileptiform activity^13^.

**Figure 2:**
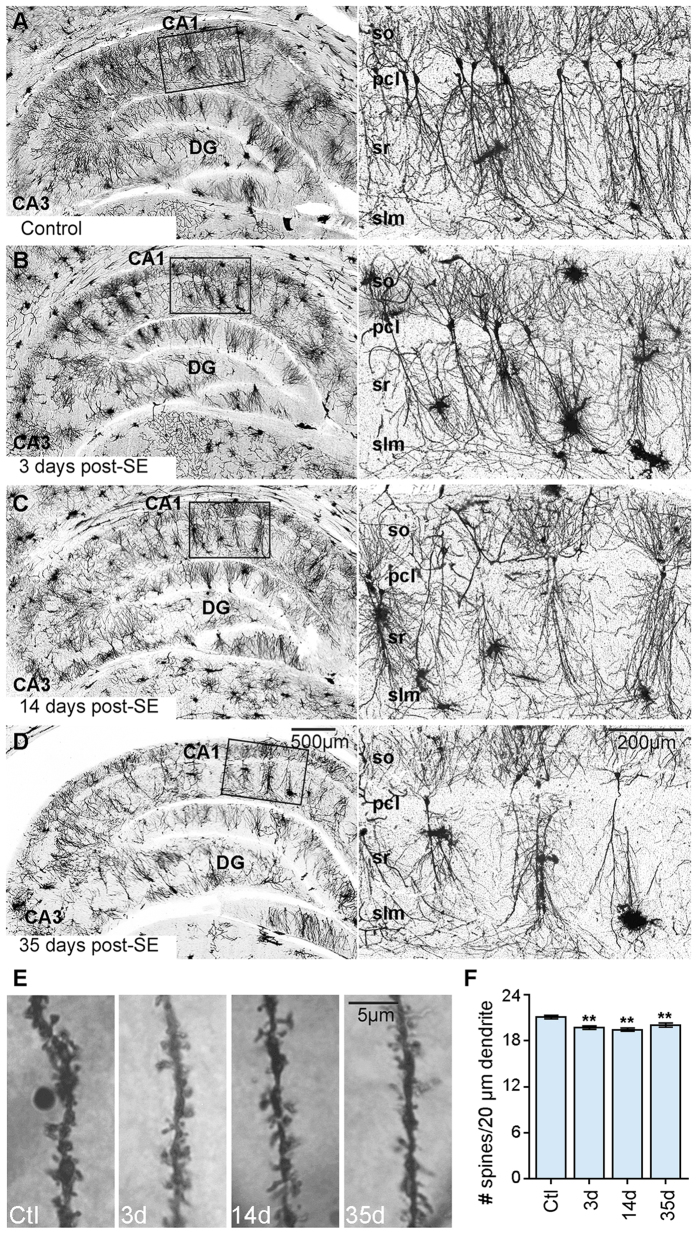
Temporal profile of changes in spine density in hippocampal CA1 cells after status epilepticus (SE). (**A–D**) shows representative images of golgi impregnated hippocampal dendrites from a control rat (**A**) and at different time points following an episode of SE ((**B**) 3 days (d); (**C**) 14d; (**D**) 35d). (**E**) shows representative images of 20 μm sections of CA1 dendritic branches from a control, and 3-, 14- and 35- days post-SE. (**F**) shows the quantitative analysis of spine density in all groups. Data are shown as mean ± standard error of the mean. **p < 0.01 compared to the control group. ANOVA with Fishers LSD post hoc test.

To determine whether dendritic arborizations were present in the CA1 region at the time points when Map2 was significantly decreased, we performed golgi staining in controls and at 3-, 14- and 35-days post-SE (Fig. 2). While golgi impregnation was evident throughout all hippocampal regions, the structural analysis of dendritic arborizations was largely obstructed by the presence of golgi impregnated glial cells in all SE groups. Thus, we quantified the spine density of second order CA1 dendritic branches and performed an ANOVA to determine a group effect [*F* (3, 1361) = 10.01, *p* < 0.01]. We found that in parallel to Map2 decline, spine density was significantly decreased at 3-, 14- and 35-days post-SE when compared to the control group (ctl vs 3d, 14d, or 35d, *p* < 0.01).

### SE triggers a transient increase in microgliosis that is prominent in the CA1 region

**Figure 3:**
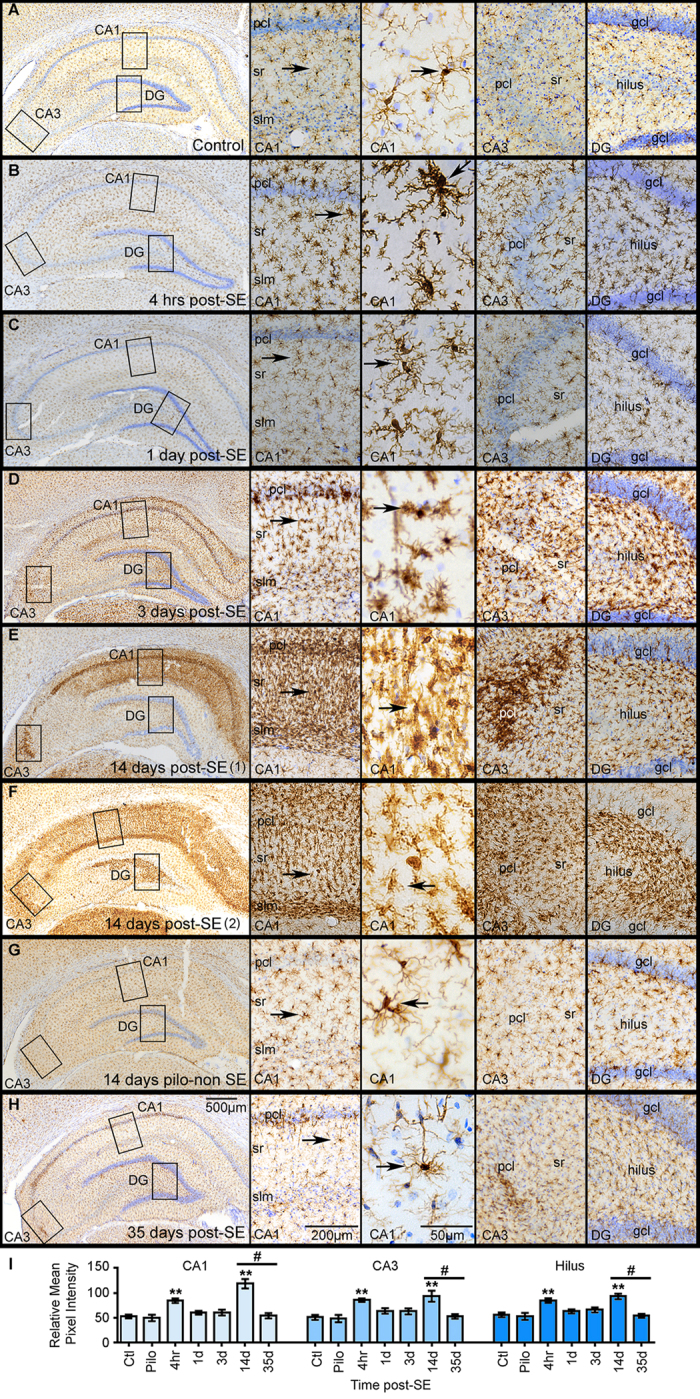
Temporal profile of IBA1 immunostaining in the hippocampus after status epilepticus (SE). (**A–H**) show representative images of the IBA1 staining (brown) from a control hippocampus (**A**) and from hippocampi collected at different time points after an episode of SE ((**B**) 4 hrs; (**C**) 1 day (d); (**D**) 3d; (**E,F**) 14d; (**H**) 35d). A representative hippocampus from a rat that was given pilocarpine but failed to develop SE (pilo-non SE; Pilo in graphs) is shown in (**G**). Right panels show high magnification images of boxed CA1, CA3, and the hilus of the dentate gyrus (DG). High magnification images with IBA1-labeled microglia (arrows) are also shown. Nissl stained cellular nuclei are shown in blue. Abbreviations: pcl, pyramidal cell layer; sr, stratum radiatum; slm, stratum lacunosum-moleculare. (**I**) shows the densitometry analysis as relative mean pixel intensity for the different hippocampal sub-regions CA1, CA3, and hilus. Significant differences in the intensity of IBA1-stained microglia are evident in the CA1, CA3, and hilar regions at 4 hrs and 14d after SE compared to the control group. Data are shown as mean ± standard error of the mean. **p < 0.01 compared to the control group. ^#^*p* < 0.05, comparison between 14d and 35d groups (n = 4–11/group). ANOVA with Fishers LSD post hoc test.

We previously showed that Map2 loss in the CA1 area correlated with accumulation of hypertrophied microglia at 2 and 3 weeks after SE^5^. However, little is known about the temporal progression between these events in the same tissues. Therefore, in parallel to Map2 and in consecutive brain sections, we mapped the temporal profile of SE-induced microglial changes in the hippocampus using IBA1 to identify this cells (Fig. 3). Densitometry analysis of IBA1 signal followed by ANOVA showed a significant group effect in areas CA1, CA3 and hilus [CA1, *F* (6, 42) = 11.28, *p* < 0.01; CA3, *F* (6, 42) = 5.99, *p* < 0.01; hilus, *F* (6, 42) = 6.22, *p* < 0.01] (Fig. 3I). In the control group, immunostaining showed a homogeneous distribution of IBA1-positive microglial cells throughout the hippocampal regions CA1, CA3, and DG (Fig. 3A). Higher magnification images from the CA1, CA3, and DG areas showed that in control hippocampi the morphological features of microglial cells included small cell bodies with highly branched and elongated processes (Fig. 3A, arrows). We found that SE triggered changes in the morphology and accumulation of IBA1-stained microglial cells in the hippocampus that progressed between 4 hrs and 35 days (Fig. 3B–H). Four hrs after SE onset, the processes of microglial cells localized throughout all hippocampal regions (CA1, CA3 and DG) were hypertrophied compared to those of control hippocampi. Furthermore, the levels of IBA1 IR at 4 hrs after SE were significantly increased throughout all hippocampal regions compared to the control group (CA1, *p* < 0.01; CA3, *p* < 0.01; hilus, *p* < 0.01). By day 1 after SE, the morphology of microglial cells and IBA1 IR levels were similar to the control group (CA1, *p* = 0.32; CA3, *p* = 0.13; DG, *p* = 0.34) (Fig. 3C). At 3 days post-SE, drastic changes were evident in the morphology of microglial cells from highly branched to amoeboid (Fig. 3D). Throughout the hippocampus, smaller amoeboid microglia displayed shortened processes at 3 days after SE compared to the earlier time points and to the control group (Fig. 3A–D). By two weeks post-SE, a robust immunostaining for IBA1-positive amoeboid microglia was concentrated within the pcl, sr, and slm of the CA1 region (Fig. 3E,F). At this time point the presence of amoeboid microglia also was evident in the CA3 pcl and in the hilar region of DG, albeit at a lesser extent to that observed in the entire CA1 area. Statistical analyses showed a significant increase in the intensity of IBA1 IR at 14 days post-SE compared to the control group in all hippocampal regions (CA1, *p* < 0.01; CA3, *p* < 0.01; DG, *p* < 0.01). The intensity of IBA1 signal in the 14 day pilo-non SE group was not different from controls in all hippocampal areas (CA1, *p* = 0.66; CA3, *p* = 0.76; DG, *p* = 0.64) (Fig. 3G). Furthermore, we found that the drastic SE-induced changes on microglial morphology and accumulation in the CA1 hippocampus were nearly resolved by 35 days after SE (Fig. 3H). Note that at this time point IBA1 IR was significantly less intense when compared to the compared to the 14 day post SE-time point in all hippocampal regions (CA1, *p* < 0.01; CA3, *p* < 0.01; DG, *p* < 0.01) and not significantly different to controls (CA1, *p* = 0.85; CA3, *p* = 0.81; DG, *p* = 0.77). Because microglial morphological changes are associated with inflammatory activation of these cells^36^, and neuroinflammation is often linked to the neuropathology of epilepsy^37^, we determined the temporal profile of a number of cytokines, chemokines, and tropic factors in the hippocampus (Supplementary Fig. S3). Consistent with several studies^37^,^38^,^39^,^40^, we found higher concentration of cytokines such as TNFα along with chemokines such as GRO/KC, MCP-1, and MIP-1α acutely after SE (4 hrs to 3 days post-SE). However, no significant differences in the levels of these inflammatory molecules were found at 14 days post-SE, when microgliosis was most prominent in the hippocampus. Taken together these data suggest that SE triggered a transient accumulation of microglia within the hippocampus that peaked at two weeks after SE and decreased thereafter.

### SE triggers a transient decrease in NeuN immunoreactivity and apoptosis in CA1 cells

**Figure 4:**
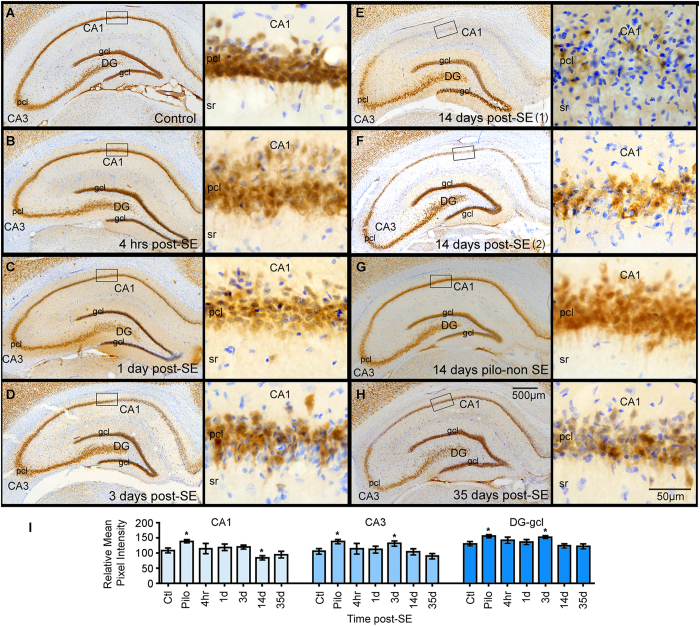
Temporal profile of NeuN immunostaining in the hippocampus after status epilepticus (SE). (**A–H**) show representative images of the NeuN staining (brown) from a control hippocampus (**A**) and from hippocampi collected at different time points after an episode of SE ((**B**) 4 hrs; (**C**) 1 day (d); (**D**) 3d; (**E,F**) 14d; (**H**) 35d). A representative hippocampus from a rat that was given pilocarpine but failed to develop SE (pilo-non SE; Pilo in graphs) is shown in (**G**). Right panels show high magnification images of boxed CA1 pyramidal cell layer (pcl) sections for each group. Nissl stained cellular nuclei are shown in blue. (**I**) shows the densitometry analysis as relative mean pixel intensity for the hippocampal CA1 pcl, CA3 pcl, the granule cell layer (gcl) of the dentate gyrus (DG). Significant differences in the intensity of NeuN-stained neurons are evident in the CA1 pcl at 14d post SE, and in the CA3 pcl and DG gcl at 3d post-SE compared to the control group. Note that the intensity of the NeuN signal in the CA1 pcl of the Pilo group is significantly elevated compared to the control group (n = 3–9/group). Data are shown as mean ± standard error of the mean. *p < 0.05 compared to the control group. ANOVA with Fishers LSD post hoc test.

Neuronal loss induced by SE may contribute to the decreased levels of Map2 as well as increased microgliosis. Therefore, to assess potential neuronal changes we used the marker NeuN to identify neurons (Fig. 4). Densitometry analysis of the NeuN signal localized within the CA1 pcl, CA3 pcl, and DG gcl showed significant group effects [CA1 pcl, *F* (6, 37) = 3.94, *p* < 0.01; CA3 pcl, *F* (6, 37) = 3.15, *p* = 0.01; DG gcl, *F* (6, 37) = 3.75, *p* < 0.01] (Fig. 4I). In controls, NeuN positive neurons outlined the regional architecture of the hippocampal principal cell layers and showed a homogenous signal within the soma of the CA1-3 pyramidal cells and the gcl (Fig. 4A). We found that at 14 days post-SE the intensity of NeuN IR was significantly decreased in the CA1 pcl when compared to controls (*p* = 0.02) (Fig. 4E,F). Albeit the levels of NeuN signal were drastically declined at 14 days post-SE, high magnification images showed that CA1 neurons contained weak NeuN IR (Fig. 4E,F). In contrast to the observations in CA1 pcl, ANOVA revealed a significant increase in the levels of NeuN IR at 3 days post-SE in the CA3 pcl and in the gcl (CA3 pcl, *p* = 0.03; DG gcl, *p* = 0.02). Unexpectedly, we also found that the intensity of NeuN signal in the hippocampal CA1 pcl, CA3 pcl and gcl of the pilo-non SE group was significantly increased when compared to controls (CA1 pcl, *p* = 0.04; CA3 pcl, *p* = 0.02; DG gcl, *p* = 0.01). At 35 days-post SE the NeuN signal was not different from the control or 14 days post-SE groups (CA1 pcl, *p* = 0.40; CA3 pcl, *p* = 0.30; DG gcl, *p* = 0.86).

**Figure 5:**
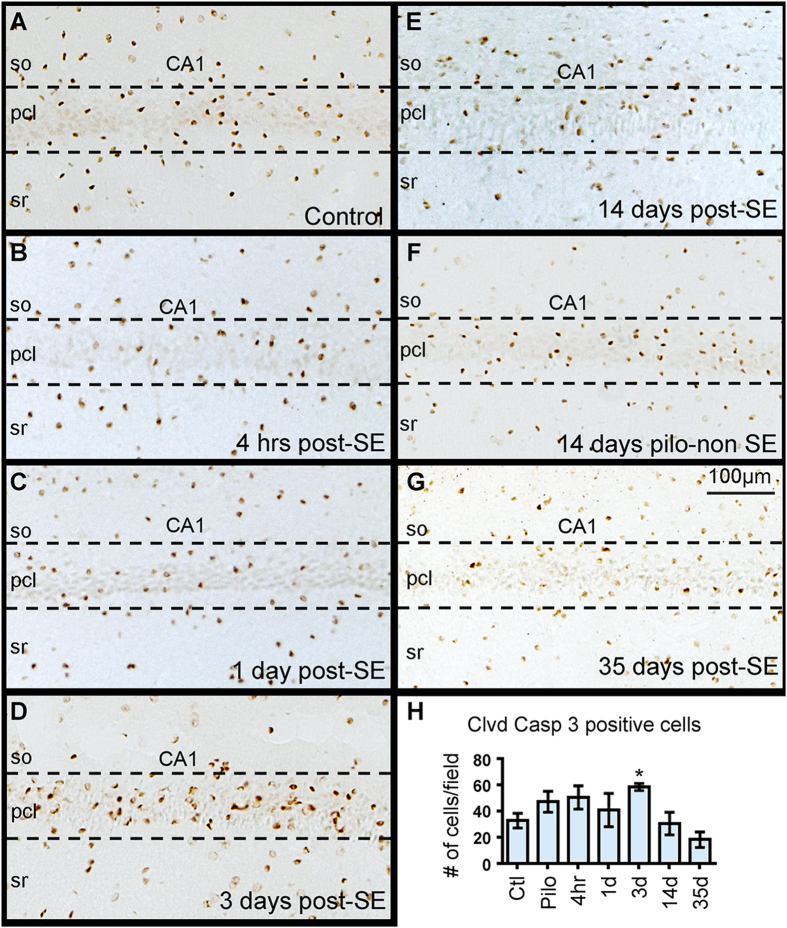
Temporal profile of cleaved-caspase 3 immunostaining in the hippocampus after status epilepticus (SE). (**A–G**) Representative images for cleaved (clvd) caspase-3 immunostaining (brown) are shown for control (**A**) and at different time points after SE onset ((**B**) 4 hrs; (**C**) 1 day (d); (**D**) 3d); (**E**) 14d; (**G**) 35d). A representative hippocampus from a rat that was given pilocarpine but failed to develop SE (pilo-non SE; Pilo in graphs) is shown in (**F**). Abbreviations: so, stratum oriens; pcl, pyramidal cell layer; sr, stratum radiatum. (**I**) Quantitative analysis of Clvd-caspase-3 positive cells show a significant increase in the number of labeled cells at 3 days post-SE compared to controls. No significant differences were evident other time points post-SE relative to controls (n = 4–8/group). Data are shown as mean ± standard error of the mean. *p < 0.01 compared to the control group. ANOVA with Fishers LSD post hoc test.

SE-induced cell loss in the hippocampus is widely reported after SE and one associated mechanism for this cell death is apoptosis^15^,^41^. Therefore, we used antibodies against cleaved-caspase 3 to identify and quantify apoptotic cells in the CA1 pcl (Fig. 5). We found a large number of cleaved-caspase 3 positive cells in controls and all SE groups and significant changes in the group comparison analysis [*F* (6, 34) = 3.54, *p* < 0.01] (Fig. 5H). The number of cleaved-caspase 3 positive cells at 4 hrs and 1 day after SE onset was not different from the control group (ctl vs 4 hrs, *p* = 0.11; ctl vs 1d, *p* = 0.46). However, at 3 days post-SE a significant increase in the number of cleaved caspase 3-positive cells was evident in the CA1 pcl when compared to controls (*p* < 0.01). Despite the drastic SE-induced loss of NeuN and Map2 IR within CA1 at 14 days post-SE, the number of cells positive for cleaved caspase-3 was not different from controls at this time point or at 35 days after SE (ctl vs 14d, *p* = 0.82; ctl vs 35d, *p* = 0.16). Taken together these data indicate that a maximal number of cells underwent apoptosis during the first week after SE and did not correlate with the temporal profile of SE-induced Map2, NeuN, or microglial changes.

## Discussion

The main findings of this study describe the spatial and temporal correlation between the SE-induced changes in Map2 IR and microglial accumulation in the hippocampal formation at various time points following an episode of SE. Specifically, we found that: (1) SE triggered a decrease in the intensity of Map2 IR in the CA1 hippocampal pcl and sr areas that was evident as early as 1 day after SE, reached minimal expression at 14 days, and was partially increased by day 35 post-SE (Fig. 1); (2) SE induced changes in the morphology of microglial cells that were evident as early as 4 hrs post-SE in all hippocampal regions. This was followed by a maximal accumulation of hypertrophied/amoeboid microglia mainly localized within the CA1 area at two weeks post-SE (Fig. 3). Even though alterations in Map2 and microglia within the hippocampal formation are often seen subsequent to SE and in epilepsy^5^,^12^,^16^,^22^,^23^,^24^,^25^,^26^,^27^,^38^, this study is the first to describe that the evolution of these events follow similar spatial and temporal profiles in an experimental model of SE and acquired temporal lobe epilepsy.

Spatiotemporal analyses of SE-induced Map2 changes in the hippocampus have been previously reported in the developing brain^16^. SE in the immature brain triggers a transient increase in the levels of the high molecular weight Map2 protein, which is the dominant Map2 isoform in the adult brain^8^,^16^. In contrast, we found that SE in the mature brain triggered a decline in the intensity of Map2 IR that was prominent in the hippocampal CA1 pcl and sr areas (Fig. 1). Studies showing that Map2 deficient mice display reduced dendritic lengths along with decreased microtubule densities^7^ suggest that the observed SE-induced Map2 loss may contribute to the altered dendritic arborizations and dendritic structural instability often seen in epilepsy^3^,^42^. Note that we found a significant decline in spine density (Fig. 2) and in the levels of phospho-Map2 IR (Supplementary Fig. 2) suggesting a correlation between these events. Since phosphorylation of Map2 at Ser136 destabilizes its association with microtubules^8^, we speculate that a decrease in the phospho-Map2 levels after SE may help stabilize microtubule assembly when Map2 levels are low.

In addition, it is possible that Map2 decline after SE may disrupt cellular processes such as dendritic trafficking^7^,^9^,^10^,^11^. Interestingly, the timeline of SE-induced Map2 changes is similar to the one described for the distribution of the dendritic HCN channels, which following SE concentrate in the somatic region of CA1 cells until at least day 30 when their localization within CA1 sr is restored^43^. While it is not known if the Map2 decline directly contributes to the altered HCN channel distribution after SE, we speculate that the transient Map2 dysregulation may contribute to homeostatic plasticity in the CA1 hippocampus. A number of studies support that when neurons are challenged with abnormal activity (i.e. seizures) their physiological responses adapt to the initial imposed changes, most likely trying to restore normal activity patterns (homeostatic plasticity)^44^. However, determining whether the transient Map2 loss is an attempt to preserve neuronal and dendritic homeostasis in response to SE, and whether it is detrimental or beneficial for neuronal stability, requires future investigation.

It is also expected that some of the SE-induced Map2 loss is a direct consequence of neuronal death and injury^12^. We found a significant increase in the number of apoptotic cells positive for cleaved-caspase 3 during the first week after SE (3 days; Fig. 5) that are consistent with a previous study^45^. However, the finding that Map2 decline was maximal at two weeks and not 3 days post-SE suggests that additional mechanisms may be underlying the disruption of Map2 expression in remaining neurons. This could be due to alterations in the activation of intracellular signaling cascades such as mTOR and MAPK/ERK pathways which are altered by SE and have been shown to regulate protein synthesis of Map2^5^,^46^,^47^. In addition, we would have expected that the intensity of NeuN IR over the CA1 pcl at 3 days post-SE (Fig. 4) would have been significantly reduced due to the increase in the number of apoptotic cells at this time point. One possibility for the lack of a significant decline in NeuN IR over CA1 pcl is that NeuN may be altered in the remaining neurons. Studies using immunofluorescence would be better suited to address potential changes in the intensity of NeuN IR in individual cells. Nevertheless, a spatial correlation between reduced Map2 and NeuN IR along with presence of reactive microglia also was reported in cortical tissue from human refractory epilepsy^14^.

Previously, we reported that prominent microgliosis overlapped with decreased Map2 IR in CA1 sr area at 2 and 3 weeks after SE^5^. In this study we showed that there is considerable spatial and temporal overlap between the progression of SE-induced loss of Map2 IR and increased microgliosis in the CA1 area (dorsoventrally and bilaterally) (Supplementary Fig. S1). Substantial evidence support that inflammatory activation of microglial cells after SE contributes to some of the neuropathological changes associated with prolonged seizures^37^,^38^. We confirmed neuroinflammation at the acute time points following pilocarpine-induced SE^39^,^40^, which correlated with early changes in Map2. In contrast, at the 14 days post-SE time point when microgliosis was most prominent (Fig. 3) no significant changes were evident in at least 20 inflammatory mediators (Supplementary Fig. S3). These data support that the initial SE-induced microglial morphological changes, including hypertrophied processes, observed between 4–24 hrs may be associated with their inflammatory activation. Microglia are phagocytic cells that clear dead cells and cellular debris along with neuronal elements such as synapses^48^. Thus, the vast accumulation of amoeboid microglia with shorter processes in CA1 pcl may be associated with a phagocytic phenotype^36^. This may be in response to the increased number of apoptotic cells. We speculate that once the dead cells within CA1 have been cleared, microglial cells recede from this area over time but not before possibly potentiating dendritic alterations in the remaining neurons.

Recent studies show that SE enhances the attraction of microglial processes toward neural elements^49^ and increases the density of cell-to-cell contacts between activated microglia and CA1 dendrites^5^,^50^. This is important because a growing body of evidence supports the idea that microglia participate in shaping neuronal dendritic and synaptic connectivity^33^,^34^,^51^. For instance, microglial processes regularly survey their surrounding microenvironment making direct contacts with spines and synaptic structures which they can engulf and eliminate^33^,^34^,^51^,^52^. Thus, it is conceivable that the vast accumulation of microglial cells in CA1 may play a role in the disruption of dendritic structures. In future studies we will investigate this possibility.

Taken together, our findings suggest that SE-induced Map2 and microglial changes mirror each other’s spatiotemporal profiles. Given recently described novel functions for microglial cells in the regulation of neuronal connectivity^33^, our findings may lay the foundation for future mechanistic experiments to identify potential roles for microglia in the modulation dendritic structures in epilepsy. In addition, a map of microgliosis and its association with other pathological SE-induced changes in the hippocampus (e.g. inflammation, astrogliosis, transcriptional and/or translational dysregulation) may lead to the identification of more specific time windows for pharmacological interventions using immunosuppressants. For instance, it is possible that the temporal profile of SE-induced microglial alterations may contribute to the discrepant observations reported following early vs. late rapamycin treatments in models of SE and acquired epilepsy^5^,^17^,^53^,^54^,^55^.

## Materials and Methods

### Animals

Male Sprague Dawley rats (150–175 grams) (Harlan Laboratories) were housed at the Psychological Sciences Building. Ambient temperature was constantly 22 °C, with diurnal cycles of a 12-hour (hr) light and 12-hr dark (8:00 to 20:00 hr). All animals had access to unlimited food and water.

### Pilocarpine-induced status epilepticus

SE was induced using previously described protocols^5^. Briefly, rats were injected with scopolamine methylbromide (1 mg/kg) intraperitoneally (i.p.). Thirty minutes (min) later, injections of saline (Control) or pilocarpine (280–300 mg/kg; Sigma Chemical Co., St Louis, MO, USA) (SE group) were administered (i.p.). SE onset was determined by development of class 5 limbic motor seizures (rearing and falling)^56^. SE was allowed to continue for up to 1 hr, at which point seizure activity was stopped with diazepam (10 mg/kg; i.p.; Sigma Chemical Co.). Two hours after, injections (i.p.) of sterile 0.9% saline (AddiPak) were administered for hydration. Sliced peeled apples and Kellogg’s Fruit Loop cereal were placed in all rats’ cages in addition to the rat chow, for up to one week after SE onset. All rats were monitored daily for adequate food/water intake and for body weight. Animals were sacrificed at the following time points after SE: 4 hrs, (n = 4), 1 day (n = 6), 3 days (n = 9), 14 days (n = 7) and 35 days (n = 7) after SE. A sham (control) group (n = 11) and a pilo-non SE group for the 14 day time point (n = 9) were analyzed in parallel.

### Immunohistochemistry (IHC)

Rats were profoundly anesthetized with Beuthanasia (200 mg/kg) and perfused with ice cold 1X phosphate buffered saline (PBS) (PBS; 137 mM NaCl, 2.7 mM KCl, 4.3 mM Na_2_HPO_4_, 1.47 mM KH_2_PO_4_, pH 7.4) followed by 4% paraformaldehyde (PFA). After overnight post-fixation (4%-PFA) and cryoprotection (30% sucrose), brains were frozen in pre-chilled isopentane, and stored at −80 °C until used for IHC. IHC was done following previously described protocols^5^,^18^,^57^. Brains were sliced in coronal sections (50 μm) using a Leica CM1860 cryostat, and stored in 1XPBS + 0.1% Sodium Azide at 4 °C. For colorimetric IHC, we used serial sections along the dorsoventral axis at approximately the following Bregma coordinates: −3.00 mm, −3.48 mm, −4.08 mm, −4.36 mm, −4.92 mm, and −5.28 mm. These sections represent an equal sampling of the hippocampus along its dorsoventral axis (see Supplementary Fig. 1). IHC was done in free floating sections. All sections were washed in 1XPBS (5 mins), incubated in 3%H_2_O_2_ (30 min) and then in 1XPBS + 3% Triton (1XPBS-3%T) (20 min). Sections were then placed in immuno buffer (5% goat serum, 0.3% BSA, 0.3% triton diluted in 1XPBS) for a minimum of 1 hr at room temperature. Then, sections were incubated overnight on a rotating platform at 4 °C with the following primary antibodies: anti-mouse Map2, anti-mouse NeuN (1:3K; Millipore, Temecula, CA); anti-rabbit IBA1 (1:3K; Wako, Cambridge, MA); and anti-rabbit cleaved caspase 3 (1:1K; Cell Signaling Technology; Boston, MA). Anti-rabbit phospho-Map2 (Ser 136) was also used (1:1K; Cell signaling) (Supplementary Fig. 2). Following a series of washes in 1XPBS-0.1%T, sections were incubated in biotinylated anti-mouse or anti-rabbit secondary antibodies (Vector labs, Burlingame, CA) (1 hr), washed, and incubated with ABC Avidin/Biotin complex solution. Following washes in 1XPBS-0.1%T, signal was visualized using the DAB Peroxidase (HRP) Substrate Kit, 3,3′-diaminobenzidine (DAB) according to manufacturer’s instructions (Vector Laboratories). Sections were mounted in gelatin-coated slides, air dried, Nissl stained, dehydrated through increasing alcohol concentrations [50%, 70%, 95%, 100%], de-fatted in Xylene, and coverslipped using Permount mounting media. All chemicals were obtained from Fisher Scientific unless otherwise indicated.

### Golgi Staining

Rats were profoundly anesthetized with Beuthanasia and perfused with ice cold 1XPBS. All brains were rapidly dissected and processed using the FD Rapid Golgi Stain kit following the manufacturer’s instructions (Neurodigitech, San Diego, CA, USA). Brains were incubated in golgi impregnating solutions provided in the kit for a minimum of 4 weeks. Then, brains were cut into serial coronal sections (80 μm thick), mounted on gelatin-coated slides, stained following the FD Rapid Golgi Stain kit protocol. After staining, sections were dehydrated through increasing alcohol concentrations [50%, 70%, 95%, 100%], de-fatted in Xylene, and coverslipped using Permount mounting media. Quantification of spine density was performed using a 100X immersion (oil) objective with a Leica DM5500 microscope equipped with a high definition Leica DFC290 camera and using the LASV4.6 software. Five representative sections were selected along the dorsoventral axis at approximately the following Bregma coordinates: −3.48 mm, −4.08 mm, −4.36 mm, −4.92 mm, and −5.28 mm. Five CA1 neurons were randomly selected per section. From these, the number of spines was counted in 20 μm sections of five second order dendrites per neuron as previously described^5^. Total dendritic branches analyzed per group: Control: 375; 3 day post-SE: 375; 14 days post-SE: 375; 35 days post-SE: 240. Brains per group: Controls: 3; 3 day post-SE: 3; 14 days post-SE: 3; 35 days post-SE: 2.

### Semi-quantitative densitometry analysis

Immunostaining was visualized using a Leica DM500 microscope and images for quantitative analyses were captured with high resolution digital camera (Leica MC120 HD) with 4X objectives using the LAS4.4 software. The relative mean pixel intensity of the immunostaining signal was acquired using the Image J NIH software (V1.49) by investigators blinded to treatment group as previously described^58^. Brain tissues that were damaged and the hippocampal anatomical landmarks were broken and thereby unrecognizable following the free-floating IHC procedures were excluded from the quantitative analyses. Therefore, between 4 and 6 sections were analyzed per brain. Densitometry analyses were performed bilaterally over the hippocampus.

### Cell counts

Semi-quantitative analyses of cells immunostained with cleaved-caspase 3 were performed by investigators blinded to treatment groups using the cell counter available in the Image J software. Images were captured using a 20X objective in a Leica DM5500 microscope equipped with a high definition Leica DFC290 camera and using the LASV4.6 software. Cleaved-caspase 3 positive cells contained within the visual field over CA1 pcl^53^ and localized inside a rectangular box (80 μm × 370 μm) were counted in all sections. Between 4 and 6 sections were analyzed per brain. Cell counts were performed bilaterally over the hippocampus.

### Statistical Analyses

IBM SPSS Statistics 22 software was used for statistical analyses and Analysis of Variance (ANOVA) with Fishers LDS post hoc tests to determine statistical significance (α < 0.05) between the control and experimental groups. Values are reported as means ± SEM. Figures were generated using Adobe Photoshop (CS6).

### Ethics Statement

All procedures concerning animals were approved by the Purdue Institutional Animal Care and Use Committee and followed in accordance to the approved Institutional and NIH guidelines.

## Additional Information

**How to cite this article**: Schartz, N. D. *et al.* Spatiotemporal profile of Map2 and microglial changes in the hippocampal CA1 region following pilocarpine-induced status epilepticus. *Sci. Rep.* **6**, 24988; doi: 10.1038/srep24988 (2016).

## Supplementary Material

Supplementary Information
