# Supplementary material for: Spatiotemporal profile of Map2 and microglial changes in the hippocampal CA1 region following pilocarpine-induced status epilepticus
Source: Sci Rep. 2016 May 4;6:24988. doi: 10.1038/srep24988 (PMC4855223; doi:10.1038/srep24988)
Supplement: Supplementary Information [file srep24988-s1.pdf]

Title: Spatiotemporal profile of Map2 and microglial changes in the hippocampal CA1 region following pilocarpine-induced status epilepticus

Author list: Nicole D. Schartz, Seth A. Herr, Lauren Madsen, Sarah J. Butts, Ceidy Torres, Loyda B. Mendez, Amy L. Brewster

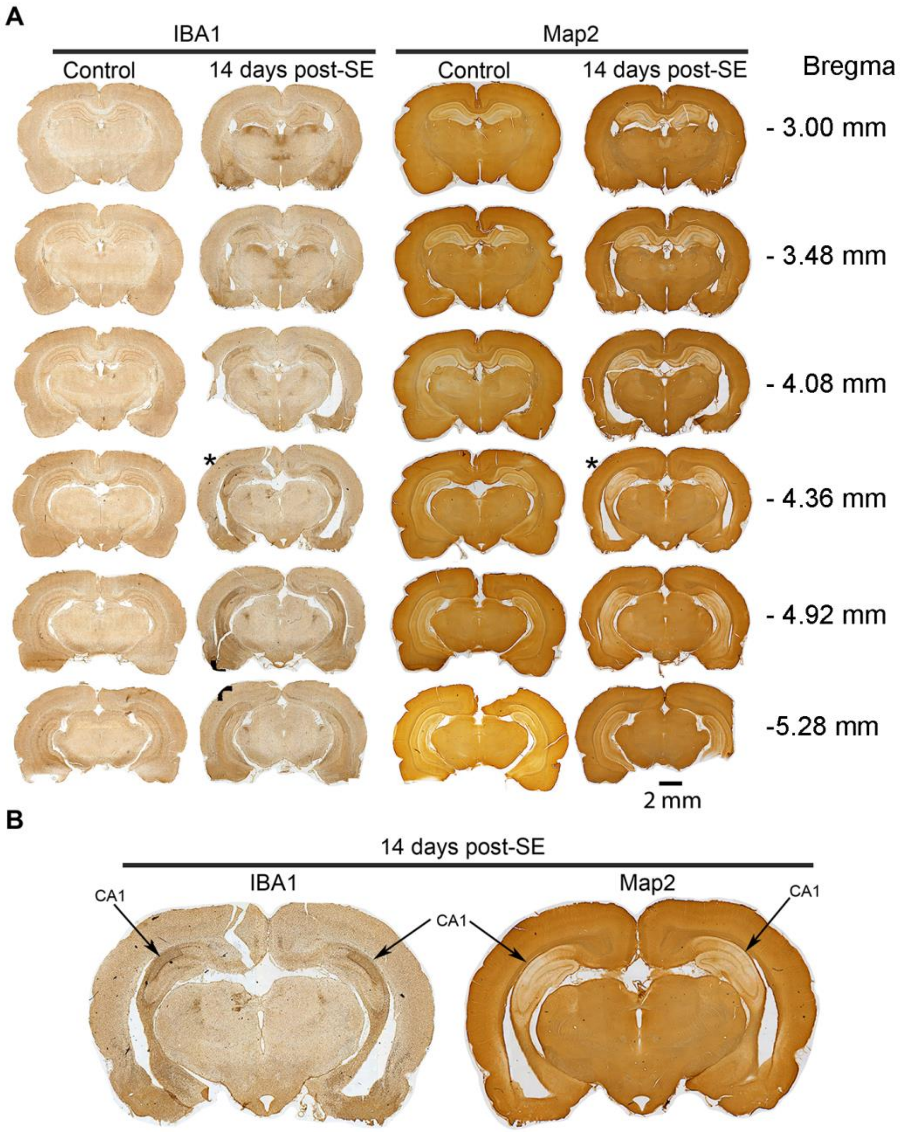

Supplementary Figure S1. **Status epilepticus (SE) induces changes in the distribution of Map2 and microglia throughout the entire CA1 hippocampus.** A, serial dorso-ventral coronal sections from a representative control brain and a 14 days post-SE brain are shown. Tissue sections were immunostained (brown) for IBA1, a marker for microglia (left panel) and Map2, a marker for dendrites (right panel). The images show that under control conditions the IBA1 staining and Map2 signal were homogeneous throughout the brain (left panels of A and B). At 14 days post-SE, the

IBA1 immunostaining in the hippocampus was more intense within the CA1 area all throughout dorsal and ventral hippocampus. Similarly, the SE-induced decline in Map2 immunoreactivity was evident bilaterally and dorsoventrally throughout the hippocampus. Approximate Bregma coordinates are shown in the far right panel. High magnification images for consecutive sections selected (*) from the 14 day post-SE series stained with IBA1 and Map2 are shown in B. Arrows, point to the similarities in the localization of intense IBA1 staining and decline in Map2 signal within the CA1 area.

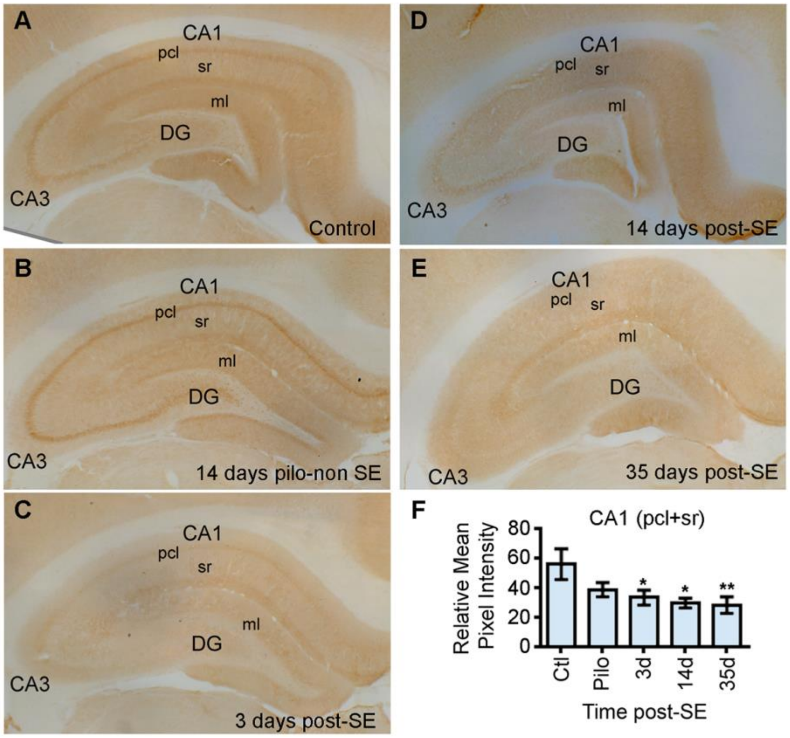

Supplementary Figure S2. **Spatiotemporal profile of phospho-Map2 immunostaining in the hippocampus after status epilepticus (SE).** A, shows a representative image of the phospho-Map2 staining (brown) from a control hippocampus. C-E show representative hippocampal images at different time points after an episode of SE (C, 3 day (d); D, 14d; E, 35d). A representative image of a hippocampus from a rat that was given pilocarpine but failed to develop SE (pilo-non SE; Pilo in graphs) is shown in B. F, shows the densitometry analysis as relative mean pixel intensity for the CA1 hippocampal sub-regions CA1 pyramidal cell layer (pcl) and stratum radiatum (sr) where total Map2 levels were significantly decreased (see Fig. 1). Significant differences in the intensity of phospho-Map2 immunoreactivity are evident within the CA1 region between the control group and 3-35d post-SE groups (n = 6-9/group). Data are shown as mean ± standard error of the mean. *, $p < 0.05$, **, $p <$

0.01 compared to the control group. ANOVA with Fishers LSD post hoc test. Abbreviations: ml, molecular layer of the dentate gyrus (DG).

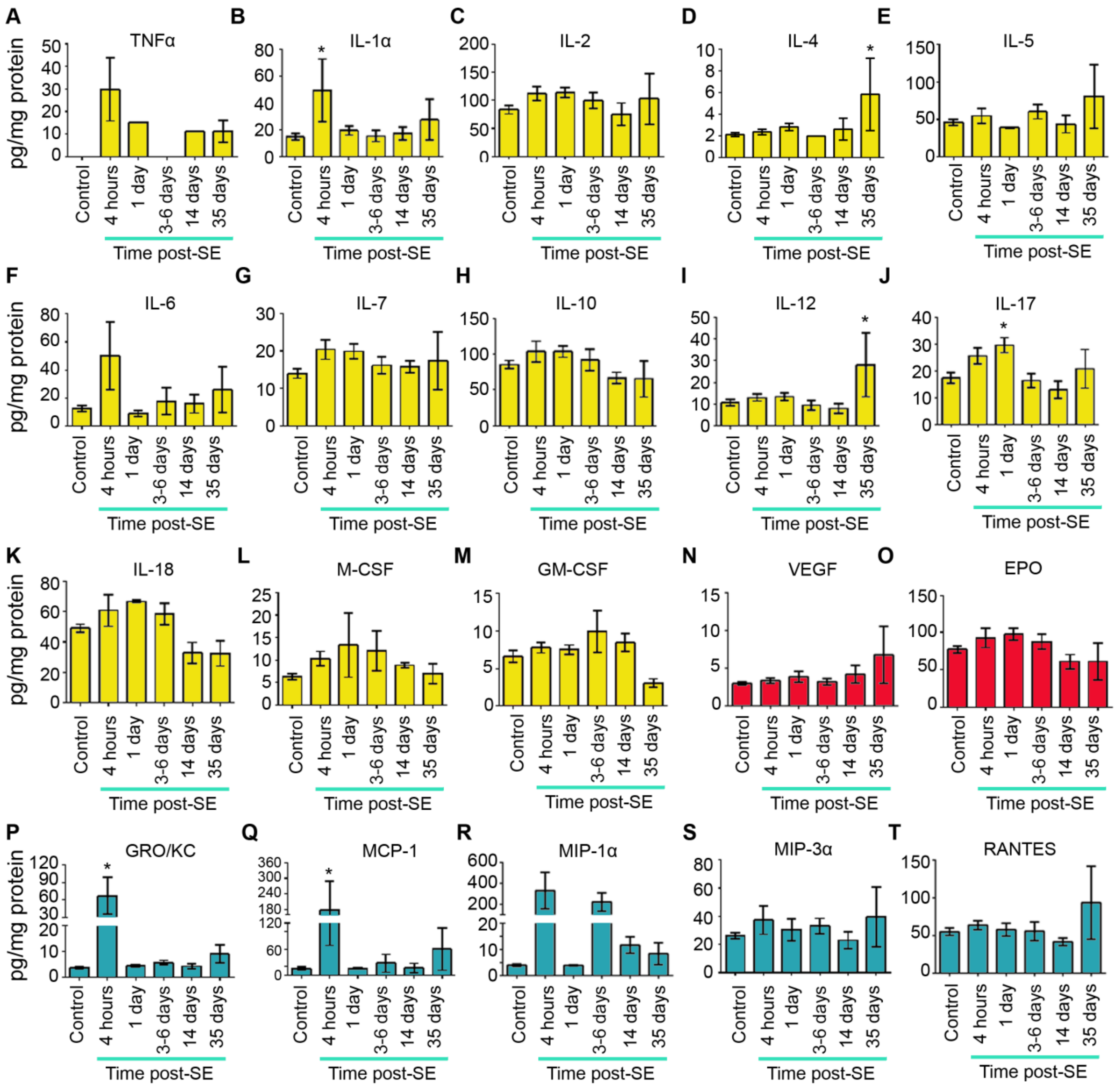

Supplementary Figure S3. **Temporal profile of inflammatory markers in the hippocampus after status epilepticus (SE).** Protein levels of cytokines (yellow bars) are shown in A-M, growth factors (red bars) in N-O, and chemokines (blue bars) in P-T. Increases in the inflammatory markers TNFα, IL-1α, IL-17, GRO/KC, MCP-1 and MIP-1α were mainly observed between 4 hours and 3-days post-SE. Note that the protein levels for cytokines such as TNFα as well as the chemokine MIPα remained largely

below detection levels in the control group. For TNFα all 10 control samples were below detection levels in the immunoassay while only 3/10 showed detectable levels of MIP-1α. Thus, the observation that high levels for these inflammatory proteins were evident at various acute time points after SE suggests an increase that is related to the prolonged seizures. The protein levels for each inflammatory molecule were normalized to total protein concentrations of the respective whole hippocampal homogenates. The protein levels were measured using a 24-plex magnetic bead-based immunoassay (Bio-Plex Pro Rat Cytokines 24-plex) with a Bio-plex MAGPIX platform according to manufacturer instructions (BioRad, Hercules, CA). N numbers are as following, controls (10); SE groups: 4hrs (5); 1 day (5); 3-6 days (6); 14 days (8); 35 days (3). Data are shown as mean ± standard error of the mean. *, $p < 0.05$, by ANOVA with Dunnett's post-hoc test (controls vs. experimental groups).
